# A neuro-inspired model-based closed-loop neuroprosthesis for the substitution of a cerebellar learning function in anesthetized rats

**DOI:** 10.1038/srep08451

**Published:** 2015-02-13

**Authors:** Roni Hogri, Simeon A. Bamford, Aryeh H. Taub, Ari Magal, Paolo Del Giudice, Matti Mintz

**Affiliations:** 1 Psychobiology Research Unit, School of Psychological Sciences and Sagol School of Neuroscience, Tel Aviv University, Tel Aviv 69978, Israel; 2 Complex Systems Modeling Group, Istituto Superiore di Sanità, 00161 Rome, Italy; 3 Istituto Nazionale di Fisica Nucleare, Sezione di Roma, 00185 Rome, Italy

## Abstract

Neuroprostheses could potentially recover functions lost due to neural damage. Typical neuroprostheses connect an intact brain with the external environment, thus replacing damaged sensory or motor pathways. Recently, closed-loop neuroprostheses, bidirectionally interfaced with the brain, have begun to emerge, offering an opportunity to substitute malfunctioning brain structures. In this proof-of-concept study, we demonstrate a neuro-inspired model-based approach to neuroprostheses. A VLSI chip was designed to implement essential cerebellar synaptic plasticity rules, and was interfaced with cerebellar input and output nuclei in real time, thus reproducing cerebellum-dependent learning in anesthetized rats. Such a model-based approach does not require prior system identification, allowing for *de novo* experience-based learning in the brain-chip hybrid, with potential clinical advantages and limitations when compared to existing parametric “black box” models.

Fast-paced research in the field of neuroprostheses places increasing emphasis on closing the loop between the brain and artificial devices. Neuroprosthetic systems were first applied to substitute sensory inputs^1^ or motor outputs^2^,^3^. More recently, neuroprostheses have been bidirectionally interfaced with the brain with the aim of detecting predefined brain signals or states to instruct brain stimulation in improving the function of motor neuroprostheses^4^, as well as ameliorating neurologic symptoms of Parkinson's disease^5^ and epilepsy^6^.

Currently, such closed-loop neuroprostheses are considered for substitution of malfunctioning central brain structures. However, examples of closed-loop systems are scarce, due to the difficulty of embodying a satisfactory model of the central nervous functional circuit in an artificial device compact enough to be conceived as a neuroprosthesis. Functional substitution of a brain circuit requires building a reliable model of its operation. The best attempt so far has targeted the hippocampus^7^, which lends its extensively studied connectivity and physiology to selective substitution of its substructures. Achieving this and extending the approach to even more complex brain circuits (like the prefrontal cortex of non-human primates^8^) was possible by adopting powerful general-purpose non-linear system-identification techniques like Volterra series, which effectively lump possibly very complex dynamics into a limited set of kernels. In this sense, these efforts could be classified as “black box” approaches.

However, there is clearly potential in trying to capture the dynamic principles at work in the brain substructures to be substituted. The good knowledge of the modular functional connectivity of the cerebellum^9^ offers an opportunity for an approach to cerebellar neuroprostheses rooted in theoretical modeling (e.g., refs. 10,11,12,13,14,15). Such models have previously been embedded in robots, allowing them to acquire and execute “adaptive” motor behaviors based on their “experiences” in physical environments^10^,^11^,^15^. Rather than applying a black box approach, such systems by and large utilize learning rules derived from putative mechanisms of the biological cerebellar system, and could thus be considered “neuro-inspired”. Learning in such robotic systems depends on artificial sensors (e.g., cameras, infra-red sensors) to provide the neuronal model with cues. Conversely, a neuro-inspired closed-loop system must rely on real, dynamic neuronal sensory representation, and could thus serve as an additional means to evaluate the performance of a model – which is by definition an incomplete representation of a neural circuit. In this respect, the current study provides additional support for the functionality of a cerebellar model previously embedded in a robotic device that successfully acquired conditioned motor responses (CRs)^10^.

Here, a similar model was bidirectionally interfaced with the brains of anesthetized rats, and reproduced cerebellum-dependent learning of the temporal relationship between a benign auditory conditioned stimulus and a noxious somatosensory unconditioned stimulus, such that brain-chip hybrids gradually acquired anticipatory CRs, the onset latency of which roughly coincided with the onset of the unconditioned stimulus. Thus, this study provides a proof-of-concept demonstration of a neuro-inspired model-based neuroprosthetic system capable of real-time experience-driven *de novo* learning without prior system identification. This is in contrast to previous black box systems, which reproduced behavior that was previously learned by the biological brain, but did not show *de novo* learning in brain-machine hybrids^7^,^8^. Importantly, the artificial cerebellar model was designed to imitate essential synaptic plasticity mechanisms underlying cerebellar learning while maintaining the necessary simplicity that would allow for it to be embedded, together with filtering and detection stages required for deciphering the cerebellar inputs, in a small and low-power VLSI chip, thereby paving the way for implantable neuroprostheses.

## Results

### The cerebellar learning microcircuit as a target for functional rehabilitation

**Figure 1:**
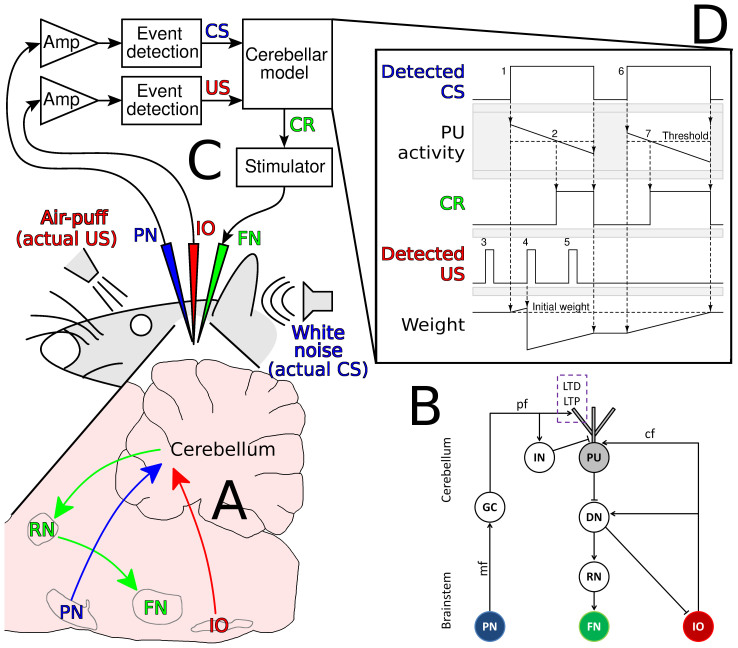
System overview. (A), A sagittal section illustrating brainstem-cerebellum input and output pathways underlying eyeblink conditioning: The pontine nucleus (PN) and inferior olive (IO) relay the auditory conditioned stimulus (CS) and somatosensory unconditioned stimulus (US) to the cerebellum, respectively. Following conditioning, a conditioned response (CR) is generated by the cerebellum, and is relayed via the red nucleus (RN) to the motor facial nucleus (FN) which elicits a blink. (B), Schematic depiction of the neural circuitry. Arrows and bars represent excitatory and inhibitory synapses, respectively. The PN-CS signal is relayed to cerebellar granule cells (GC) via mossy fibers (mf), and then through parallel fibers (pf) to both Purkinje cells (PUs) and inhibitory interneurons (IN). The IO-US signal is relayed to PUs via climbing fibers (cf). Convergence of CS and US signals on a PU causes LTD at the pf-PU excitatory synapse (CS-PU_E_; dashed rectangle). If only the CS signal arrives at the PU the CS-PU_E_ synapse undergoes LTP. PUs regularly inhibit deep cerebellar nuclei (DN) neurons, and DN disinhibition results in elicitation of a conditioned blink via the FN, as well as IO inhibition. (C), Brain-chip interface. Recording electrodes were implanted in the PN and IO, to detect CS and US events during eyeblink conditioning. Eyeblink CRs were elicited via a stimulating electrode implanted in the FN. (D), Performance of the cerebellar model during two trials. Detection of CS onset (1) triggered a slowly decaying PU response, with a starting level proportional to the weight of the synthetic CS-PU_E_ synapse; when PU activity dropped below a threshold (2) it triggered a CR; CS detection resulted in LTP expressed as an increase in CS-PU_E_ synaptic weight; US events not coinciding with CS detection caused no change in synaptic weight (3); concomitant CS and US detection (4) resulted in LTD, expressed as a reduction of CS-PU_E_ synaptic weight; when CRs overlapped with US events, the latter were masked, simulating IO inhibition by the DN, driving LTP rather than LTD (5), which, in the subsequent trial (6), defined the starting amplitude of the PU trace and the delay to threshold crossing (7).

**Figure 2:**
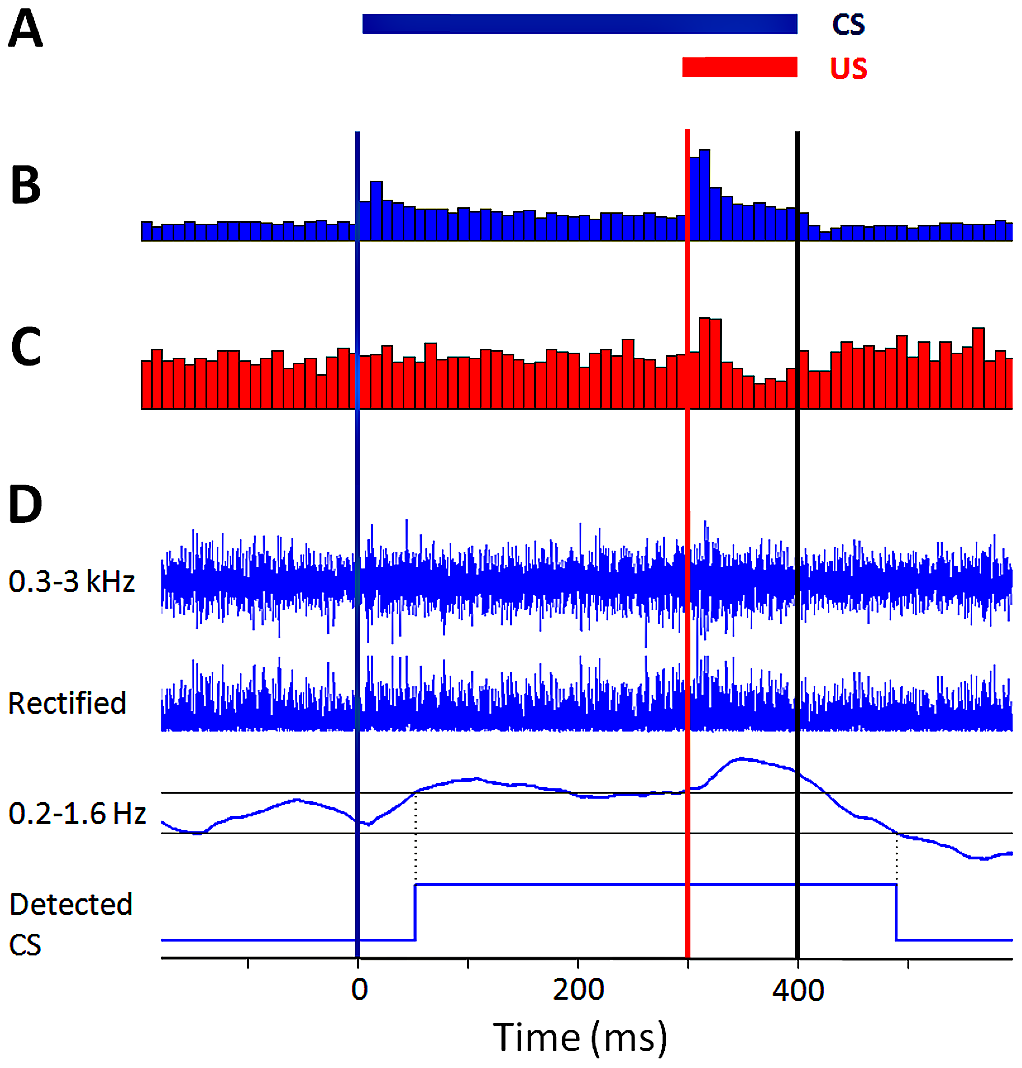
Event detection. (A), Paired CS (blue; 400 ms) and US (red; 100 ms) presentation. Onsets and offset (black) are shown as vertical lines in b-d (time 0 = CS onset). (B–C), Multiple-unit activity recorded from the PN (B) and IO (C) of hybrid #20 was rectified and thresholded (3*mean), and peri-stimulus time histograms (PSTHs) were created from multiple trials (56 in this example) to determine and characterize reactivity to stimuli. This data was then used to parameterize on-chip event detection for the real-time experiment. (D), Single-trial event detection example. The signals from the PN electrode were amplified, band-pass filtered (0.3–3.0 kHz) and the 3 channels were summed to yield the top trace; this was rectified (second trace), and then band-pass filtered (0.2–1.6 Hz; third trace); hysteretic thresholds (two gray horizontal lines) were then applied to yield the detected CS event in the bottom trace; the delay of onset detection, here ~50 ms, is due mainly to the time taken to aggregate information before making a decision, a consequence of the choice of filter band; the hysteretic threshold captures the duration of the CS event (after the onset delay) without detecting false alarms from the smaller threshold incursions. Additional examples of individual trials and further response analyses are presented in Supplementary Fig. 3.

To present reliable indices of functional recovery, we chose to experiment with a learning function localized in a discrete brain microcircuit. This requirement is met by the cerebellar microcircuit learning to time a discrete movement (Fig. 1). This function is often tested by employing the eyeblink conditioning paradigm, consisting of repeated trials comprised of a conditioned stimulus (CS) paired with an unconditioned stimulus (US) - typically an auditory CS preceding a periorbital-airpuff US by several hundred ms (Fig. 2a), and by monitoring the acquisition of eyeblink-CRs triggered by the CS^9^,^16^. The necessary microcircuit is located in the cerebellar hemispheres, lesions of which permanently prevent and abolish eyeblink conditioning^9^,^17^. Conditioned motor responses are not expressed under general anesthesia^18^ (see also Supplementary Figs 1 and 2a), and therefore the observed motor responses of the brain-chip hybrids would depend on deep brain stimulation controlled by the on-chip synthetic cerebellar circuit, rather than on biological compensation mechanisms or spared cerebellar function that would be expected in surgical or chemical lesion studies.

Cerebellar conditioning follows well-known dynamics, which provide reliable indices by which to evaluate the performance of brain-chip hybrids. Initial eyeblink-CRs are infrequent and not adaptive in the sense that they tend to follow, rather than precede, the onset of the expected US. As learning progresses, CR rate increases and CR onset latency decreases^19^,^20^,^21^ (but see ref. 22). Eventually, CR rate stabilizes at an asymptotic level, substantially <100% in rats, with the peak of CR amplitude adaptively coinciding with the US's onset^16^,^21^,^23^. Subsequent delivery of CS-alone trials (i.e. unpaired with USs) leads to gradual extinction of CRs. This learning paradigm is therefore suitable for demonstrating gradual, experience-related plasticity in a synthetic neuroprosthesis, strongly correlated with its output behavior.

### Construction of the neuro-inspired cerebellar model

The challenge was to construct a prosthetic chip which would acquire and perform anticipatory CRs by relying on essential mechanisms of the biological cerebellum. The cerebellar model was previously described^10^,^24^. Briefly, in each acquisition trial, CS and US signals are relayed through the brainstem pontine (PN) and inferior olive (IO) nuclei, respectively^19^, and converge on specific Purkinje cells (PUs) in the cerebellar cortex. When no stimuli are presented, spontaneous PU activity inhibits the output neurons of the deep cerebellar nuclei (DN)^25^,^26^. When a CS is presented, it drives PU activity through excitatory (CS-PU_E_) and inhibitory synapses^27^,^28^ (Fig. 1b). During the initial conditioning trials, PU neurons respond with continuous firing, and thus inhibit the output DN neurons throughout the entire CS-period. The concurrent arrival of the US signal to PUs causes long-term depression (LTD) of CS-PU_E_ synapses^29^,^30^. The result is trial-by-trial reduction in the net excitatory drive, causing a gradually shorter PU response to the CS signal, followed by an absolute pause in PU firing for the rest of the CS period - or until the US signal arrives at the PU. DN neurons thus experience gradually earlier disinhibition, to which they respond with elevated activity which excites motor pathways, thus triggering a motor-CR^21^,^31^,^32^.

In conclusion, in our model the learning-related LTD in the CS-PU_E_ synapses is responsible for the following cascade of events: reduction in the net excitation of PUs by the CS; shortening the delay from CS onset and the CS-evoked pause in PU activity; shortening the delay to DN disinhibition; and finally, shortening the onset latency of motor-CRs until they precede the US onset in a well-conditioned organism^19^,^20^,^21^. The DN also exerts an inhibitory feedback on the IO^32^,^33^ (see Fig. 1b). In the final stage of learning, when DN disinhibition results in an anticipatory CR, the elevated DN activity is also appropriately timed to inhibit the US signal at the IO level, thus blocking the US signal from reaching the cortical PU^33^,^34^,^35^; in these trials, only the CS signal reaches the PU, causing long-term potentiation (LTP) of CS-PU_E_ synapse. In subsequent trials, this LTP increases net PU excitation and thus delays DN disinhibition until DN-IO inhibition is once again too late to block the US signal at the IO level, reestablishing LTD at the CS-PU_E_ synapse. This cyclic process stabilizes DN disinhibition and CRs in an oscillatory manner, such that the CR onset latency jitters around the predicted time of US onset while CR rate is kept below 100%. CS-alone trials cause LTP, which results in extinction of the CR by the aforementioned mechanism.

To adjust the neuro-inspired model for learning the CS-US association in real-time, the circuitry programmed onto a field-programmable chip^24^ was reduced to its most basic elements (Fig. 1d). The combined effect of CS-evoked excitation and inhibition of PUs was modeled as a descending trace of PU activity. When this trace dropped below a certain (arbitrary) threshold, it was considered to represent a PU pause, disinhibiting the DN; note, however, that prior to learning, CS presentation never caused the PU trace to cross this threshold. The chip acquired and processed the raw multi-unit activity recorded from the rat's PN and IO (Fig. 2 and Supplementary Fig. 3) and extracted the CS and US events, respectively, in real-time (Fig. 2d). The detected CS event was directed to a single excitatory CS-PU_E_ synapse while the weight of this synapse defined the starting amplitude of the descending PU trace. If a US was detected during the CS period, synaptic weight was decreased, representing learning-related LTD, such that in subsequent CS detections, the starting amplitude of the PU trace would be lowered; if only a CS was detected, synaptic weight was increased, representing extinction-related LTP; if only a US was detected, no synaptic change was induced.

The cerebellar chip did not include an explicit DN component that would generate excitation of motor pathways. Rather, capitalizing on the dependence of the DN disinhibition on the level of CS-PU_E_ synaptic weight, a threshold PU response level was defined, below which detected CSs triggered eyeblink CRs by applying an electrical train to the facial motor nucleus. When this train overlapped with would-be detection of a US event it generated electrical artifacts which masked US-evoked IO activity. Therefore, USs could not be detected for 150 ms following CR onset, and therefore could not induce LTD. Thus, masking of the US signal by the electrical artifact served as an effective implementation of DN disinhibition-related blocking of the US signal at the IO level. In this respect, the present system differs from its predecessors^10^,^24^, in that it does not include a built-in delay in DN-IO inhibition, in the order of tens of ms^33^. As a result, following CR acquisition, CR latency in the hybrids would be expected to stabilize such that CR onset would roughly coincide with the onset of the US. This is in contrast to the intact animal, in which CR onset precedes the US by 100–200 ms, such that the CR reaches its peak amplitude around the time of the expected US^20^,^22^.

### Interfacing the prosthetic chip with cerebellar inputs and output

Embedding the prosthetic chip in its biological milieu required its interface with the inputs and outputs of the cerebellar microcircuit underlying eyeblink conditioning. The cerebellar inputs are well-mapped, and accordingly, auditory-CSs and airpuff-USs were extracted from multiple-unit records from the PN and IO, respectively, both considered immediate precerebellar nuclei^19^,^26^. Occasionally, US events were extracted from records in the sensory trigeminal nucleus upstream of the IO^36^, to evaluate the performance of the hybrid under improved US detection conditions. Eyeblink-CRs were triggered by delivering electrical trains to the facial nucleus (See Supplementary Figs 2b and 4) or to the zygomatic branch of the facial nerve downstream of the facial nucleus^37^.

### Learning by the hybrid system

The progress of learning was tested in anesthetized rat-chip hybrids during acquisition (paired CS-US trials; inter-stimulus interval = 300 ms) and extinction (CS-alone trials). In hybrids #20 and #21, recording electrodes were placed in the precerebellar PN and IO nuclei and stimulating electrodes were placed in the motor facial nucleus. Fig. 2b–c shows peri-stimulus time histograms of hybrid #20's PN and IO responses to paired CS-US trials delivered during the parameterization stage (see Methods) – i.e. prior to the acquisition block, during which the cerebellar chip did not undergo any plasticity and the hybrid showed no eyeblinks. The PN responded both to the CS and to the US, consistent with the sensory converging properties of the PN^38^, with a phasic component of ~25 ms in duration, followed by a sustained period of elevated activity lasting throughout the duration of both stimuli. The IO responded selectively to the US with a phasic component of 15–20 ms in duration, comparable with IO responses previously observed by us and others^39^,^40^. To test the performance of the hybrid under more favorable US detection conditions, in hybrid #15 the recording electrodes were placed in the PN and in the trigeminal nucleus, upstream of the IO^36^, where a larger proportion of true positive US detections was observed (see below). During chip parameterization (see Methods), LTD and LTP magnitudes were set to produce CRs with latencies ≈300 ms within 60 trials^24^ (note that a physiologically realistic number of trials would be 300–500 in the rat^21^,^23^, and a few tens of trials in humans^20^).

**Figure 3:**
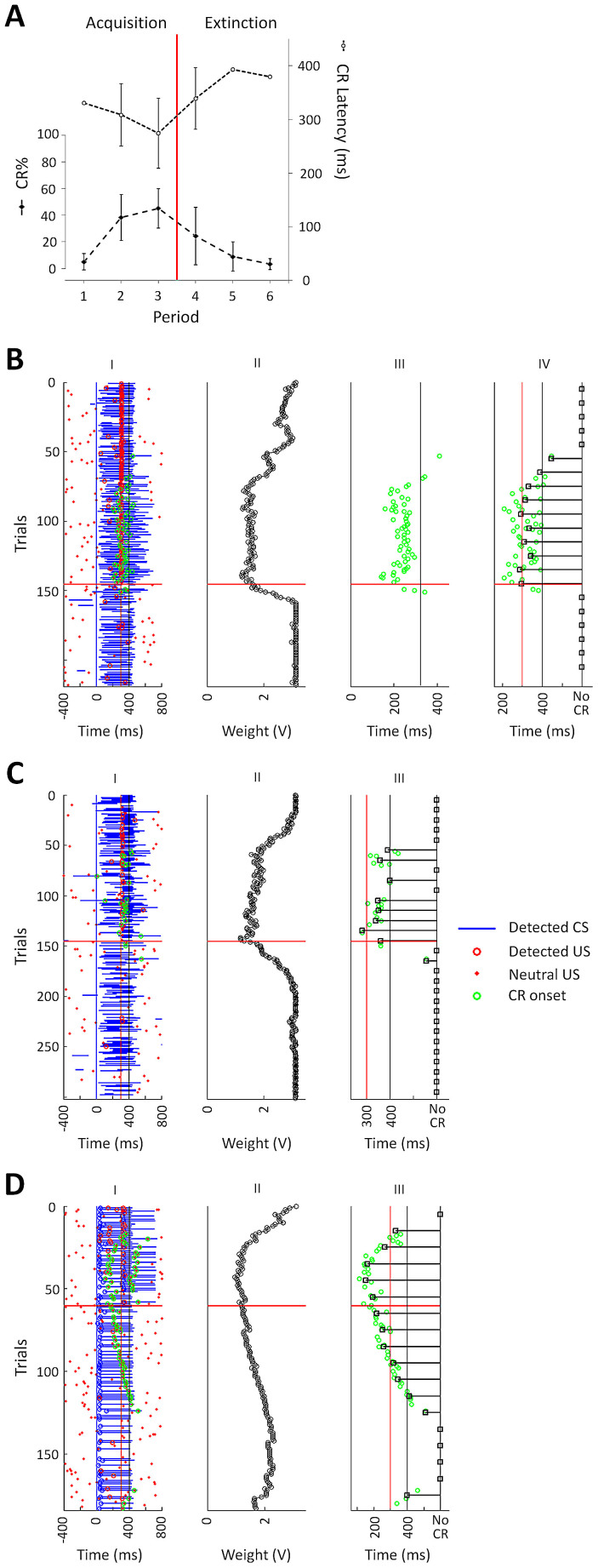
Conditioning in brain-chip hybrids. (A), Progression of learning across all hybrids (n = 3; mean ± s.e.m.). Only CRs triggered by true CS detections are depicted. CR rate increased during acquisition and decreased during extinction; CR onset latency was negatively correlated with CR rate. Points without error bars indicate that this value was derived from a single hybrid (#21) – as other hybrids exhibited no CRs during these periods, and were not included in calculating the correlation between CR rate and latency. (B–D), Trial-by-trial data for each hybrid. Vertical blue, red and black lines indicate CS onset (time 0), latency to US onset, and co-termination of both stimuli, respectively. Acquisition (top) and extinction (bottom) blocks are separated by a red horizontal line. US detections outside periods of detected CSs or following a CR did not induce LTD (see Figure 1c), and are marked as “neutral”. (B), Hybrid #15. Acquisition: 145 paired CS-US trials. Extinction: 73 CS-alone trials. (I): Stimuli detections and produced CRs. (II): The weight of the synthetic synapse (represented as an analogue voltage) dropped gradually during acquisition, and stabilized from around trial 80 onwards; in the extinction block it rose back to its maximum value within 15 trials. (III): CR onset latencies relative to detected CS onsets. The vertical black line is the median of detected CS offsets. CR onset latency followed the synaptic weight trajectory. (IV): CR onset latencies relative to actual CS onset, for CRs produced by true CS detections (within 150 ms from CS onset). The larger variability in CR latencies compared to III is the result of variable CS detection delay. Black squares show results by groups of 10 trials. (C), As in b(I, II and IV), but for hybrid #20. Acquisition: 145 trials; extinction: 156 trials. (D), As in c, but for hybrid #21. Blue circles indicate that the PN response recorded in this hybrid was phasic, and therefore CS detection was assigned a fixed duration of 400 ms. Acquisition: 60 trials; extinction: 123 trials. During the last extinction period, false US detections during the CS period caused a recurrence of CRs.

Fig. 3a shows the progression of learning throughout acquisition and extinction across hybrids (n = 3). To allow for better comparison between hybrids, the number of trials per learning block (acquisition and extinction) was divided into 3 equal periods. As expected by system design, CR rate increased with the number of paired trials during the acquisition block, and decreased as CS-alone trials were delivered during the extinction block (block*period interaction, F(2,4) = 9.2, P = 0.03; repeated measures ANOVA). In addition, throughout learning, CR rate and CR latency were negatively correlated (r = −0.75, P = 0.01; one-tailed Pearson's test).

The results obtained from hybrid #15 offer the best example of the learning process in a single hybrid (Fig. 3b). The weight of the CS-PUE synapse gradually decreased along acquisition trials; the first eyeblink CR appeared in the 53rd trial, and in subsequent trials CR latency shortened (Fig. 3b, III and IV). During the last period of acquisition, CR rate reached 45.2%, and CR onset grossly coincided with the US onset (mean ± s.e.m of CR latency = 313 ± 0.01 ms). During the extinction block, synaptic weight rose to its original value within 15 trials, with the last CR observed in the 5th extinction trial (trial 150 overall). In hybrid #20 (Fig. 3c), the first CR appeared in the 56th trial, and thereafter learning was less effective than in hybrid #15, with CR rate and latency reaching 22.5% and 342 ± 0.01 ms, during the last period of acquisition, respectively. During the extinction block, synaptic weight rose to its original value within 40 trials, with the last CR observed in the 17th extinction trial (trial 163 overall). In hybrid #21 (Fig. 3d), CRs were acquired faster than expected, with the first CR appearing in the 17th trial. During the last period of acquisition, CR rate and latency were 50% and 169 ± 0.01 ms, respectively. During the extinction block, synaptic weight initially rose, but slower than expected, and did not reach its original value. Moreover, after 81 extinction trials, synaptic weight began to decline, and during the last extinction period a 10% CR rate was observed (as compared to 0% in hybrids #15 and #20).

Deviations from the expected progress of learning – as predicted during chip parameterization, were presumably the result of inconsistent detection of CS and/or US events (see also Supplementary Fig. 5). Since LTD depended on concomitant CS and US detection, misdetections of either stimulus would obstruct acquisition; CS misdetection would prevent expected LTD, while US misdetection would promote LTP. Conversely, false detection of either or both stimuli would increase the chance of concomitant detection, resulting in excessive LTD, obstructing extinction. To assess the quality of event detection in each hybrid, we examined the True Positive Proportion (TPP) and False Positive Rate (FPR) for both CS and US detections from PN and IO/trigeminal records, respectively (Supplementary Fig. 6). TPP was defined as the proportion of trials in which a stimulus was detected within a predefined time window. FPR was defined as the rate (in Hz) in which stimuli were detected outside of the TPP window. The width of the CS detection window was set at 150 ms, to allow for robust detection on one hand and a minimal interval of 150 ms between CS and US detection on the other hand – consistent with the minimal inter-stimulus interval allowing for robust cerebellar learning in the awake animal^9^,^22^. The width of the US detection window was set at 60 ms, since IO population responses are typically phasic, peaking within this period^40^.

In hybrid #15, CS TPP was 71%, and CS FPR was 0.1 Hz; US TPP and FPR were 93% and 0.57 Hz, respectively. In hybrid #20, CS TPP and FPR were 40% and 0.1 Hz, respectively, and US TPP and FPR were 29% and 0.23 Hz. As compared to hybrids #15 and #21, hybrid #20 showed the lowest TPP for both stimuli, leading to relatively poor acquisition. In hybrid #21, CS offset could not be reliably detected, due to a reduced sustained component of PN activity; thus the CS was considered to last for a fixed duration (= 400 ms). CS TPP and FPR were 55% and 0.02 Hz, respectively, and US TPP and FPR were 43% and 0.96 Hz. The high US FPR led to excessive LTD which, during acquisition, caused properly detected CSs to elicit CRs with onset latencies that were shorter than the pre-defined latency range, and also contributed to slow and incomplete extinction in this hybrid.

We cannot exclude the possibility that conditioning resulted in plasticity in the cerebellum, pre-cerebellar recording sites, or upstream of them (e.g. ref. 41). However, such plasticity could only affect the performance of the brain-chip hybrids by altering the neuronal signals recorded from the pre-cerebellar nuclei during conditioning. Analyses of event detection throughout conditioning sessions did not reveal any systematic effect of time or learning stage on detection quality that could have been driven by biological plasticity (Supplementary Fig. 6). Thus, it seems unlikely that such plasticity would have contributed to successful learning in the hybrids. Importantly, due to anesthesia, rats did not exhibit any blinking that was not a result of electrical stimulation of motor pathways, while stimulation parameters were set to reliably produce eyeblinks (Supplementary Figs 2b and 4). Thus, the hybrids' motor responses (i.e., eyeblinks) were exclusively controlled by the neuroprosthesis.

## Discussion

In this proof-of-concept study, we sought to integrate existing scientific and technical knowhow in developing a closed-loop neuroprosthetic system capable of reproducing a cerebellar learning function. An artificial cerebellar synapse was implemented as an analogue circuit in a VLSI chip. Neuronal signals from precerebellar nuclei were continuously fed to the chip and processed in real time to extract CS- and US-evoked responses, which instructed learning in the synthetic synapse. The weight of the artificial synapse determined both the rate and latency of eyeblink CRs, which were elicited by the chip via electrical stimulation of motor pathways downstream from the cerebellum. The on-chip implementation was based on a neuro-inspired model of a cerebellar microcircuit. The design, construction and application of the model could be realized for a number of reasons. First, cerebellar micro-connectivity has been mapped well enough for bottom-up modeling, while essential physiological functions have been studied at all levels of the cerebellum, allowing for top-down modeling^10^,^11^,^12^,^13^,^14^,^15^. This allowed us to capture, albeit in a simplified form, the cerebellar mechanisms essential for the function to be substituted (the effective dynamics of PU activation, and the LTD/LTP at the CS-PU_E_ synapse). Second, the sensory- CS and US inputs and the motor-CR output of the cerebellum follow distinct anatomical pathways^9^,^17^, which enabled bidirectional brain-chip interfacing. Finally, the replaced cerebellar microcircuit acquires a discrete motor-CR^35^, the learning curve of which was used to constrain the model's parameters. The neuroprosthesis reproduced the acquisition and extinction learning functions in a real-time *in-vivo* context, consistently with the theoretical model on which our design was based^10^,^24^.

The growing interest in closed-loop brain-chip systems is motivated by their potential contribution to basic science and translational advantages^42^. As mentioned in the Introduction, previous implementations of central neuroprostheses were based on “black-box” models^7^,^8^ - i.e. parametric models of the input-output relationship for a circuit to be replaced. We contrast such implementations with our neuro-inspired model-based approach, as each approach has distinct advantages and limitations that should be considered in the context of prospective clinical developments. Neuro-inspired neuroprostheses may be limited by incomplete anatomical and physiological mapping or by the diffuse localization of the function to be replaced, while black-box models can compress input-output relationships for very complex circuitry without explicit knowledge of its elements. Black-box models depend on system identification, requiring extensive a-priori sampling of training input-output signals from the intact system. This has the advantage of reproducing individual-specific adaptations - e.g. retrieval cues for memory-related tasks previously learnt by the individual^7^,^8^, enabling rehabilitation of the cognitive behaviors that are directly based on these adaptations. However, it is difficult to envision situations motivating a preemptive characterization of the model of a given brain structure in view of a forthcoming disease, while when the brain structure to be substituted is already damaged, the opportunity to exploit an individualized model determination would be substantially reduced. Moreover, existing parametric models are incapable of learning new adaptations to support new behaviors. Conversely, a neuro-inspired model could potentially replace already damaged tissue - although with a generic functional substrate lacking pre-existing individual experience-based adaptations, and support the acquisition of new behaviors. In neuropsychological terms, the hybrids based on existing parametric black box models could be considered to display symptoms of anterograde amnesia disorder, whereas the hybrids based on the present neuro-inspired model could be considered to display symptoms of retrograde amnesia disorder. Clearly, a closed-loop system that could both capture existing adaptations and acquire new ones would be clinically advantageous.

Closed-loop neuroprostheses share some technological challenges with other brain-machine interface systems, including detection of functionally-relevant neuronal signals for chronic periods. We based detection on a multi-unit signal, which provides a more stable population signal but may be less selective than single units. The real-time *in-vivo* setup brought about uncontrolled non-stationarities in the recorded neuronal signals, affecting signal processing and event detection, and consequently varying rates of acquisition and extinction. An autonomous adaptive parameter setting strategy (e.g., ref. 43) would be a desirable development for the future. On the stimulation side, the closed-loop system is prone to lose periods of input data due to contamination by stimulation-induced artifacts, especially when prolonged, high-frequency electrical trains are used^5^; a solution will require either enhanced signal-processing to clean the input data or stimulation modes that do not introduce electrical artifacts, such as optogenetic stimulation^6^.

Another common issue is how to utilize neural data to drive biologically-relevant behavior. Here, only one very simple behavior was generated by the closed-loop system, and so the present system should clearly not be considered to fully restore cerebellar function. Moreover, to allow for a robust interface between the brain and a small, low-power and potentially implantable VLSI chip, the system was implemented using a highly-simplified cerebellar model. For instance, our model contained a single plastic parallel fiber-PU synapse whereas similar learning in the biological cerebellum possibly relies on plasticity occurring in hundreds of thousands - perhaps even millions of such synapses across multiple PU neurons, as well as on plasticity in other types of cerebellar synapses, which likely contributes to the fine-tuning of CRs (for reviews, see ref. 44, 45). Furthermore, our system did not contain an explicit DN component, whereas it has been suggested that PU-DN control may follow complex rules based on the synchrony of inhibitory PU-DN inputs, which are also subject to plasticity^46^,^47^. The complexity of the driving forces affecting the DN, which constitutes the sole cerebellar output in motor conditioning, is most likely critical for the finely-timed and graded control of motor pathways^12^. Therefore, it should be noted that while the present system reproduced cerebellar-like behavior in the sense that the brain-chip hybrids learned to produce anticipatory CRs based on the CS-US interval, CR kinematics were not controlled by our system. Rather, the chip's output was a step function with a fixed duration and amplitude, and plasticity in the synthetic synapse only affected the probability and timing of step initiation. Thus, our simple neuro-inspired system can only be considered as a proxy for the to-be-replaced neuronal circuit. It does, however, provide a demonstration that a model of a specific element of the circuit to be substituted can be embodied in a chip and integrated into the real-time dynamics of its neural context. We envisage that incremental implementation of richer and more refined neuro-inspired models, bi- (or multi-) directionally interfaced with the brain, would promote a more systematic examination of theoretical models, as well as more robust rehabilitation of behavior.

The current study was performed in deeply anesthetized animals, and therefore all observed behavior (i.e., blinking) was driven by the neuroprosthesis and there were no movement artifacts that could potentially disrupt the neuronal records. While this setup allowed us to examine the feasibility of recovering behavior using the current neuroprosthetic architecture, it has obvious limitations. For instance, the increased baseline activity and reduced synchronicity in the PN of the awake rat^48^ is expected to result in less robust PN reactivity to tone and lower proportion of correct detections as compared with the anesthetized preparation. Similarly, IO activity evoked by spontaneous motor activity and by sensorimotor information related to unconditioned motor responses^49^,^50^ could potentially obstruct the detection of US-evoked IO activity, as well as complicate the temporal representation of the US, affecting CR latency. Hence, future studies in awake animals with cerebellar lesions or deteriorated cerebellar function would be necessary to both demonstrate robustness with respect to sensory and behavioral interferences, and to advance the brain-based features of the neuroprosthesis, e.g. recovering more behaviorally complex cerebellar functions that would presumably rely on similar anatomical and physiological architecture^51^,^52^, or incorporating knowledge concerning bidirectional communication of the cerebellum with other brain areas such as the amygdala and neocortex^23^,^41^,^50^,^52^,^53^,^54^,^55^,^56^, the input-output functions of which are likely to be affected by anesthesia (e.g. refs. 57, 58).

In conclusion, a single VLSI chip contained the core circuitry needed to go from raw multi-unit input activity, through filtering, event detection, implementation of synaptic plasticity, and triggering of the CR. We have gone beyond our previous demonstrations^10^,^24^ by having the chip operating autonomously in real time, taking signals from and returning stimulation to the living brain to close the loop and produce *de novo*, experience-based learning in the hybrid. While the present findings should not be considered as proof that the cerebellar model we implemented accurately mimics biological cerebellar learning mechanisms, they do demonstrate that this model can perform *in-vivo*, and thus provide a feasibility demonstration that qualifies our system as a precursor to an autonomous, implantable cerebellar neuroprosthesis.

## Methods

A system performing offline learning based on stored neuronal data was previously described^24^. The following is a summarized description of a similar system used in the current on-line closed-loop study.

### Animals

All procedures were approved by the Tel Aviv University Animal Care and Use Committee (P-07-017), and carried out in accordance with Israeli law and institutional guidelines. Experiments were performed in naïve 3 month old male Sprague Dawley rats, anesthetized with xylazine and ketamine hydrochloride (5 mg/kg and 100 mg/kg, respectively, i.p.) and head-fixed in a stereotaxic apparatus. Supplementary doses of anesthetics were administered throughout to maintain a deep state of anesthesia based on the absence of flexion responses to hind-paw pinches. Consequently, blinks (spontaneous, unconditioned, or conditioned) were never observed during the experiments unless elicited by the neuroprosthesis.

### Electrophysiology and experimental setup

The experimental setup is illustrated in Fig. 1c. Classical delayed eyeblink conditioning was employed in anesthetized rats. The paired CS-US trials were delivered at either fixed (4 s; hybrid #20) or randomized (4–8 s; hybrids #15 and #21) inter-trial interval. The CS was a 400 ms long white-noise (70 dB), with a 10 ms rising/falling gate, delivered to the right ear through a hollow ear-bar of the stereotaxic head holder. The US was 100 ms long periorbital airpuff, co-terminating with the auditory-CS, delivered through a nozzle positioned ~2 cm from the right cornea, with a pressure of 1.5 bar at the source. Stimulus delivery was controlled by a Power1401 mkII lab interface and Spike2 software (CED, UK).

Neuronal activity was recorded from the PN and IO precerebellar nuclei, which have been shown to relay the CS and US signals to the cerebellum, respectively^19^. In order to sample the spiking activity (output) of large populations of neurons from these nuclei, multiple-unit activity was recorded. Multiple-unit activity was recorded from the left PN using 3 twisted platinum wires (each with an internal diameter of 0.17 mm, ~120 kΩ), and from the left IO using a single tungsten electrode (5 MΩ, A-M systems, USA); in hybrid #15, the tungsten electrode was positioned in the trigeminal nucleus instead of the IO. Neuronal signals were amplified (10 k) and band-pass filtered (0.3–3.0 kHz) online (MCP-plus, Alpha Omega, Israel), and digitized at 15 kHz (Power1401 mkII, CED, UK). To determine the presence of an evoked response, multiple-unit activity was rectified and thresholded (3 times the average amplitude), and peri-stimulus time histograms were created (Fig. 2b–c). In order to generate eyeblink-CRs, stimulating electrodes (2 twisted platinum wires, ~120 kΩ) were implanted in the right facial motor nucleus, with final location determined by verifying an eyeblink response to a 150 ms long electrical train of pulses (0.1 ms, 200–300 μA delivered at 80–140 Hz; Grass SD9, Grass Inst., USA).

Experiments consisted of chip parameterization, followed by conditioning consisting of acquisition and extinction blocks. Parameterization began with recording PN and IO responses to paired CS-US trials in anaesthetized animals. Responses were used to parameterize the chip's detection of the CS and US events and the amplitude of LTD/LTP steps at the CS-PU_E_ synapse, using bespoke procedures implemented in MATLAB (Mathworks, USA). Typically, it was necessary to re-parameterize the chip when the records showed high variance over time; however, this was never done while a learning session was in progress. Conditioning followed with the parameterized chip attached bidirectionally (i.e., in a closed-loop) to the inputs and output of the animal's cerebellum. Paired trials were applied and the chip sampled the inputs from the PN and IO channels, detected the CS and US events and processed the simulated cerebellar LTD/LTP step on each trial, and at its output the chip controlled the timing of eyeblink-CRs by triggering electrical stimulation of the facial nucleus. The acquisition block continued until the simulated synapse reached a minimal value and remained stable for several trials, and was immediately followed by the extinction block. Extinction trials were identical to acquisition trials, except that no USs were presented.

### Histology

Following conditioning, marking lesions were made by passing anodal direct currents through the electrodes for 10 s (1 mA for PN and facial nucleus, 0.5 mA for IO). Rats were perfused transcardially with 9% formalin. Brains were removed, sliced into 30 μm coronal sections, and stained with thionine blue. Sections were examined under a light microscope to determine the final location of electrodes.

### Signal processing and chip design

Computations were implemented by a field-programmable mixed-signal array on a bespoke VLSI chip (Supplementary Fig. 7). Filters were constructed based on switched-capacitor circuit elements and amplifiers. The model was constructed using the same elements, plus digital logic where appropriate. All processes were implemented in parallel and clocks were provided by independent oscillator elements.

The chip received amplified and filtered data from the MCP-plus amplifiers. For the multi-channel electrode in the PN, the signals were summed according to a weighting calculated following offline parameterization. Neuronal signals were rectified and band-pass filtered by the chip (0.2–1.6 Hz for the PN, and 1–6.4 Hz for the IO; these bands were chosen heuristically, and were varied between experiments) to yield a measure monotonically related to the energy over a small window of time (the energy envelope). Then, thresholds were applied to detect CS and US onsets, and in some cases also the CS offset.

## Author Contributions

R.H., S.A.B. and A.H.T. contributed equally to this work, as did P.D.G. and M.M. All authors designed the project and wrote the manuscript. S.A.B. designed the VLSI chip. R.H., A.H.T. and A.M. produced the neurophysiological data used to design and implement the closed-loop brain-chip interface, and together with S.A.B. performed the closed-loop experiments.

## Supplementary Material

Supplementary Information Supplementary Figures
