# Supplementary material for: A neuro-inspired model-based closed-loop neuroprosthesis for the substitution of a cerebellar learning function in anesthetized rats
Source: Sci Rep. 2015 Feb 13;5:8451. doi: 10.1038/srep08451 (PMC4327125; doi:10.1038/srep08451)
Supplement: Supplementary Information — Supplementary Figures [file srep08451-s1.pdf]

A Neuro-inspired Model-based Closed-loop Neuroprosthesis for the Substitution of a Cerebellar Learning Function in Anesthetized Rats

Roni Hogri, Simeon A. Bamford, Aryeh H. Taub, Ari Magal,

Paolo Del Giudice, and Matti Mintz

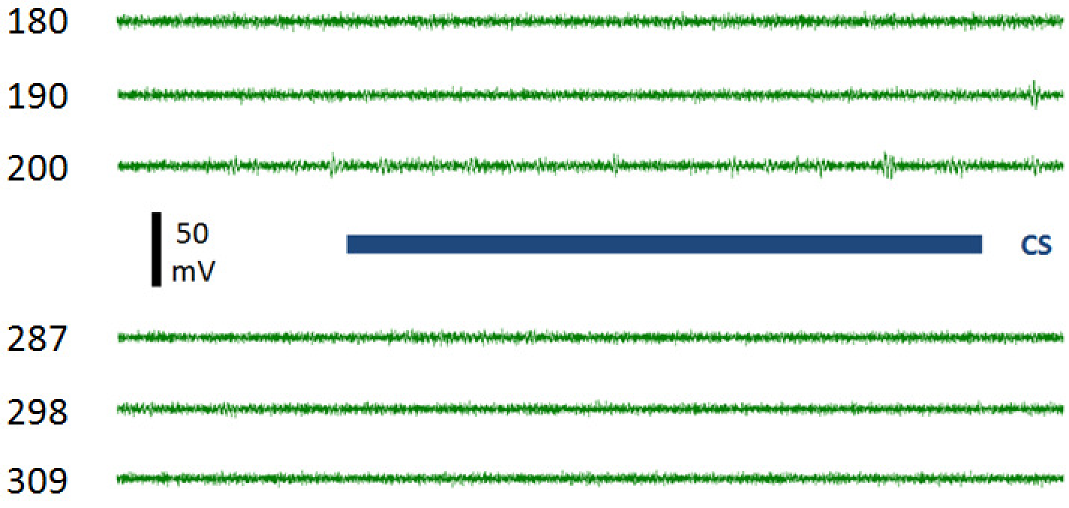

Supplementary Figure 1: Absence of CRs in anesthetized rats. EMG recorded from the orbicularis oculi of an anesthetized rat not connected to the neuroprosthesis. This rat received 20 "acquisition" blocks, each consisting of 9 CS-US paired trials and one CS-alone trial (a total of 200 trials), and 10 "retention" blocks, each consisting of 9 CS-facial nucleus stimulation-US trials, one CS-alone trial and one CS-US trial (a total of 110 trials). The EMG traces presented here are from the last 3 CS-alone trials of the acquisition block (trials 180, 190 and 200) and the last 3 CS-alone trials of the retention block (trials 287, 298 and 309). The blue bar represents the period of the auditory-CS (400 ms).

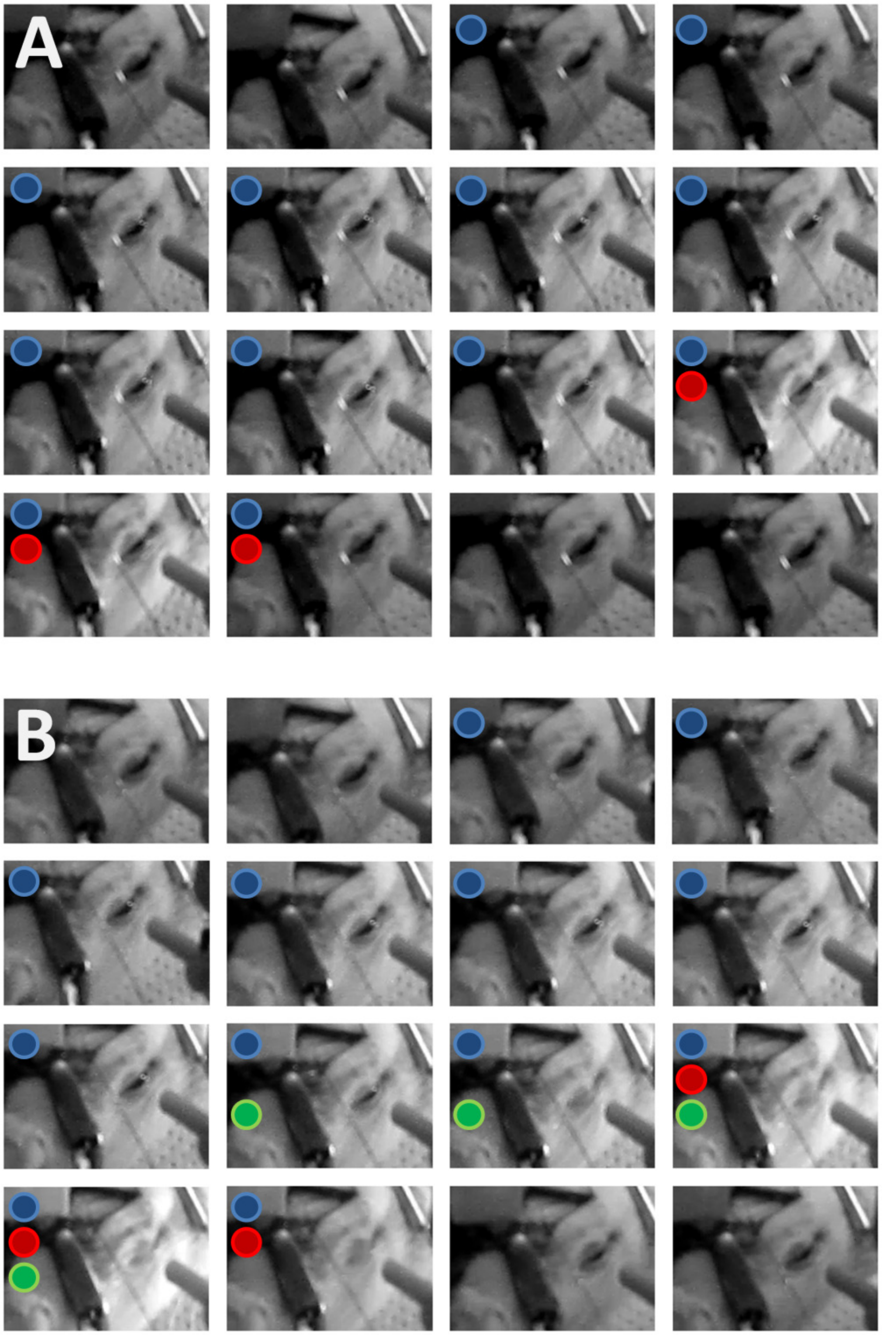

Supplementary Figure 2: Video images of behavioral responses to the auditory CS, peri-orbital airpuff US, and electrical stimulation of the facial nucleus. Frame-by-frame images of a rat's peri-orbital area (nose facing the upper right corner), recorded at 30 frames per second with a digital camera (Canon PowerShot A3300 IS). Video images were recorded during a test session which followed the

acquisition and retention blocks described in Supplementary Fig. 1. The times of stimuli was visually determined by lights activated simultaneously with stimulus delivery, controlled by a Power1401 mkII lab interface and Spike2 software (CED, UK). **a,** When no FN stimulation was given, the rat did not blink in response to the CS or the US (blue and red dots, respectively). **b,** When an electrical train (12 pulses with a duration of 0.1 ms and an amplitude of 200μA, at 80 Hz) was delivered to the facial nucleus (green dots), the rat produced a robust blink which lasted ~140 ms (see also Supplementary Fig. 4).

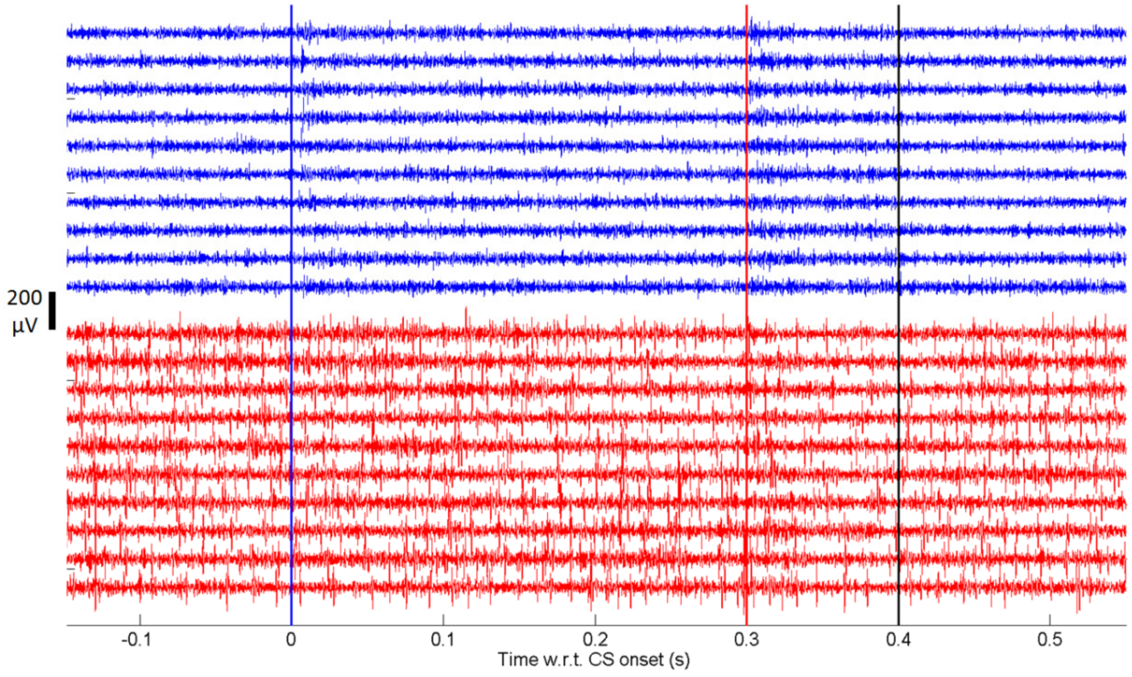

Supplementary Figure 3: Neuronal responses of the PN and IO. Representative multiple-unit activity traces recorded from the PN (blue traces) and the IO (red traces) of hybrid #20 during 10 paired CS-US trials, recorded as part of the parameterization stage of the experiment (see Methods). The blue, red and black lines represent CS onset, US onset and their co-termination, respectively. Neuronal signals were amplified and band-pass filtered (300-3000 Hz; MCP-plus, Alpha-Omega, Israel), and digitized at 15 kHz (Power1401mkII, CED, UK). Our goal was to acquire multi-unit activity, allowing for prolonged (potentially chronic) recording sessions while still observing the expected PN and IO response profiles. Therefore, we set low thresholds (mean*3 of baseline activity) to identify periods of increased energy. In this record, the mean rate of PN events was significantly higher during the 300 ms following CS onset as compared to baseline (196 versus 147 Hz); and the mean rate of IO events was significantly higher (135 Hz) and lower (57 Hz) as compared to baseline (78 Hz; all *P*s < 0.001; paired samples t-test) 10-30 ms and 30-130 ms following airpuff onset, respectively. The peri-stimulus time histograms of this recording session are shown in Fig. 2b-c.

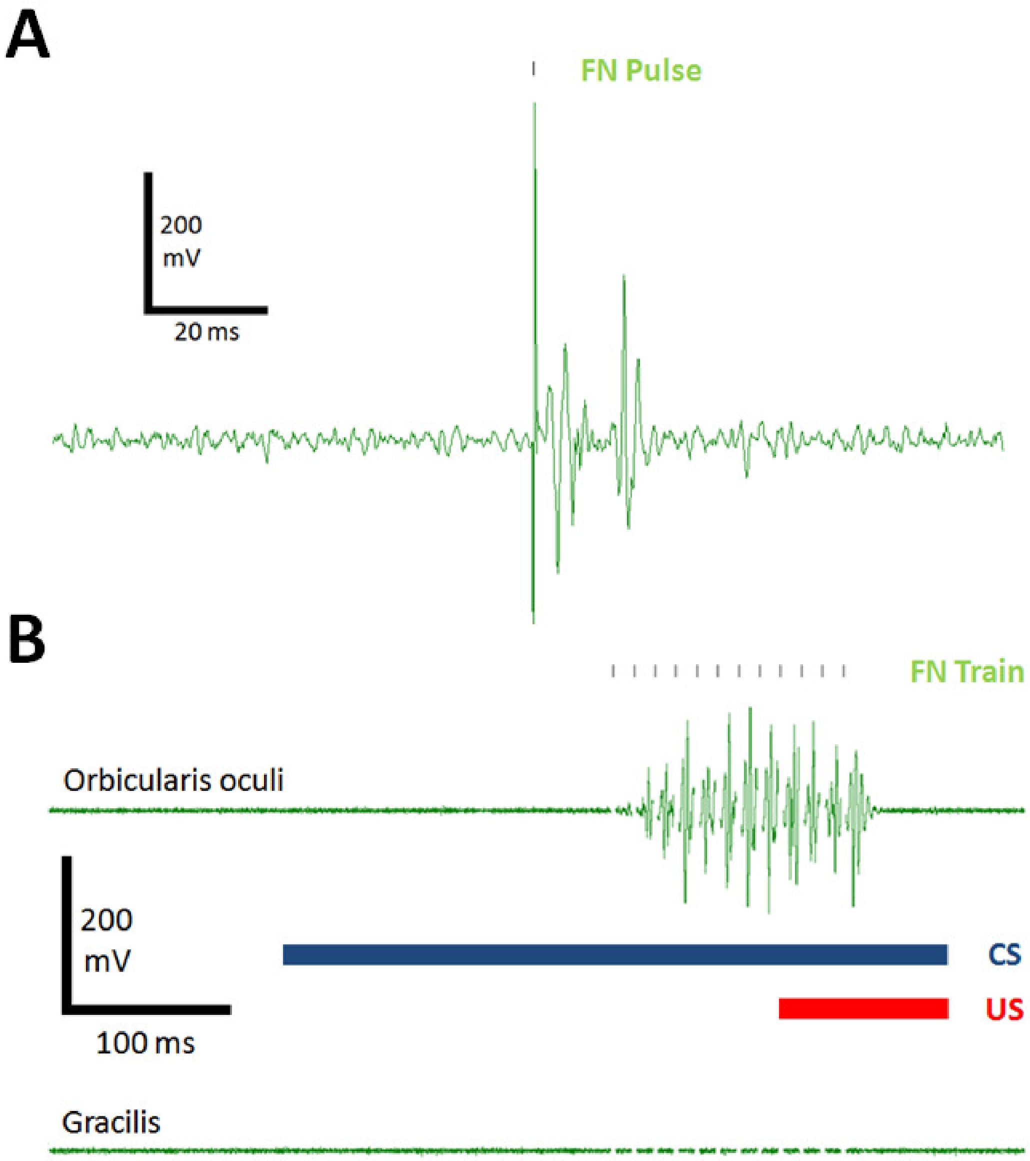

Supplementary Figure 4: EMG responses to electrical stimulation of the facial nucleus in anesthetized rats. To identify the parameters of facial nucleus (FN) stimulation necessary to reliably produce a behaviorally-relevant eyeblink-CR (a gradual and robust closure of the eyelid that would protect the animal from the onset of the airpuff-US), EMG activity was recorded from the orbicularis oculi of anesthetized rats receiving FN stimulation of varying durations, amplitudes and frequencies. **a,** In some animals a single high-amplitude pulse (300μA, 0.1 ms in duration; stimulation pulse artifact

marked by a single tick) could elicit a short-latency (~2.5 ms) EMG response with a duration of 10-20 ms. However, in all animals robust blinks necessitated trains of pulses with amplitudes ranging between 200-300μA and frequencies of 80-140 Hz. **b,** EMG recorded simultaneously from the orbicularis oculi and gracilis muscles during a CS-FN stimulation-US trial. The CS and US periods are represented by the blue and red bars, respectively. The FN stimulation train consisted of twelve 0.1 ms pulses (marked by ticks) with an amplitude of 200μA, delivered at a rate of 80Hz. Here, stimulation artifacts were removed from the EMG signals (3 ms around the peak of each pulse). The electrical train resulted in a sustained elevation in the orbicularis oculi EMG signal beginning ~20 ms after train onset and lasting until ~15 ms after the last stimulation pulse. Under this stimulation regime, robust eyeblinks were observed in 100% of trials containing FN stimulations. These eyeblinks were often accompanied by mild activation of other facial muscles (not shown). As expected, the EMG signal recorded from the gracilis muscle of the hind-limb was unaffected by FN stimulation.

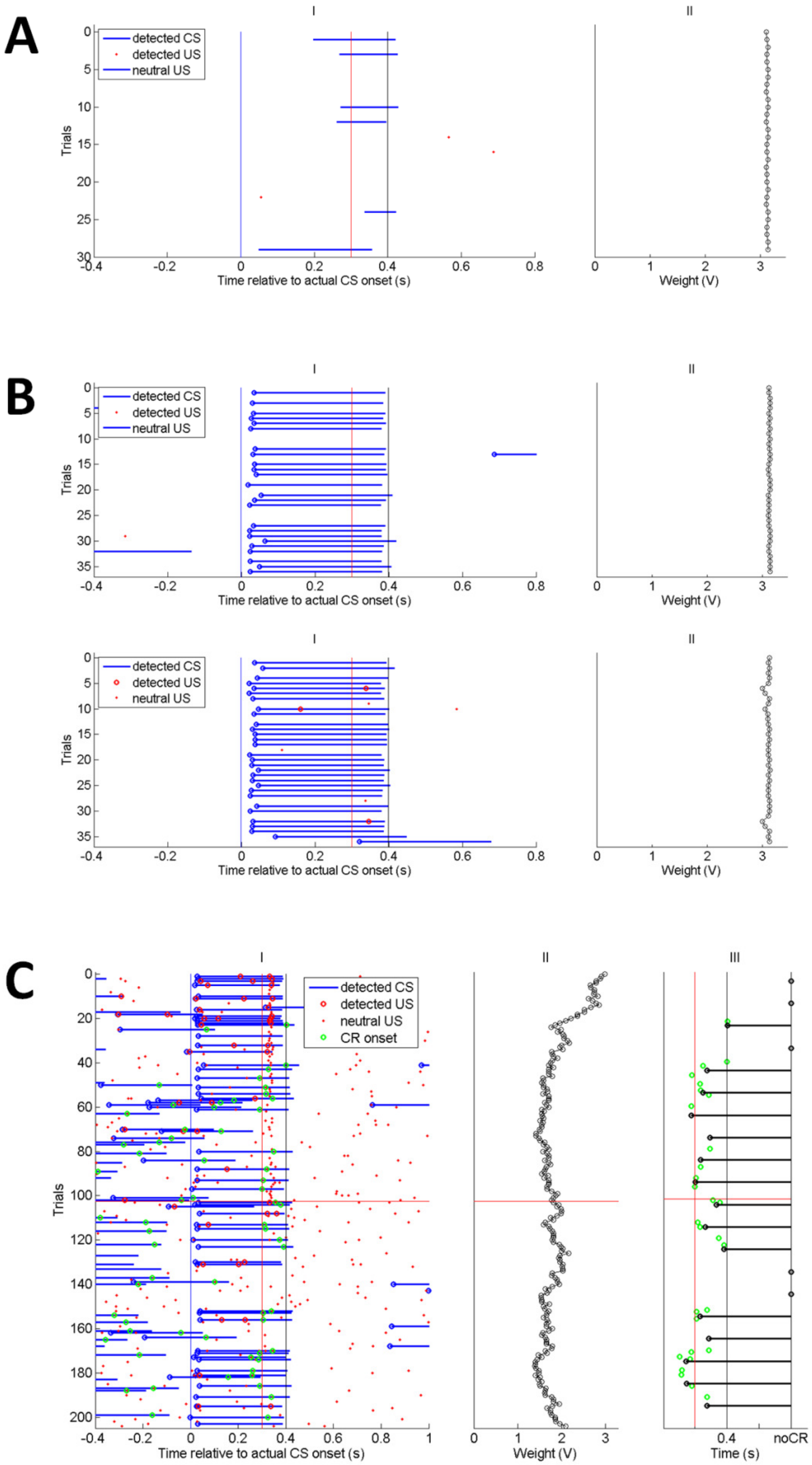
A
B
C
I
II
III
detected CS
detected US
neutral US
CR onset
Trials
Time relative to actual CS onset (s)
Weight (V)
Time (s)
noCR

Supplementary Figure 5: Consequences of deficient stimulus detection. Since anesthetized rats do not exhibit motor CRs (see Supplementary Fig. 1) the rate and latency of CRs observed in the rat-chip hybrids were completely dependent on the performance of the event detection and cerebellar model modules of the VLSI chip (see Fig. 1b and Supplementary Fig. 6). Failure to correctly parameterize event detection algorithms resulted in erroneous synaptic processes (compare with Fig. 3). **a,** High rates of misdetections of both CS and US events. The CS and US events were never detected simultaneously (I), and thus the weight of the synthetic synapse did not change at any point (II). **b,** Two examples of high rates of misdetected US events. Top: no simultaneous detection of CS and US, resulting in no change in the weight of the synthetic synapse. Bottom: in a few trials the simultaneous detections of CS and US events caused LTD, but in most trials only the CS was detected, resulting in LTP and zero net change in the weight of the synthetic synapse after 36 paired CS-US trials. **c,** High rate of false detections of both CS and US events. Most changes in synaptic weight observed here occurred irrespective of the actual CS and US. Rather, LTD was mostly driven by simultaneous false detection of CS and US, while LTP was mostly driven by false detections of the CS. As a result, most CRs were produced in inappropriate times (without CS presentation), and the presentation of >100 CS-alone trials failed to induce extinction (III). The blue, red and black vertical lines in I and III represent CS onset, US onset, and their co-termination, respectively.

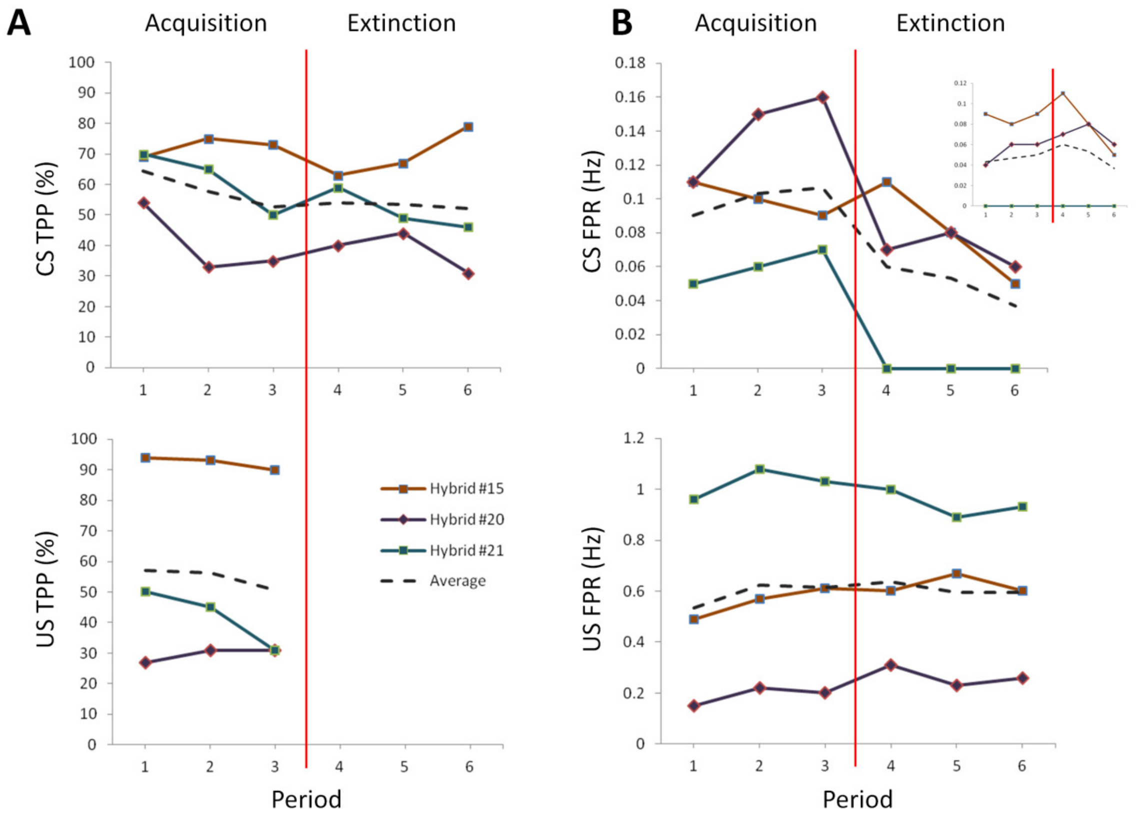

Supplementary Figure 6: Non-stationarities in event detection. For each hybrid, acquisition and extinction blocks were divided into 3 equal periods each. Variability in the true positive proportion (TPP; a) and false positive rate (FPR; b) of both CSs (top) and USs (bottom) was observed both between hybrids and throughout learning sessions. For US TPP calculation, only trials in which there was no risk of the IO's response to the US being masked by the ~150 ms electrical artifact induced by CR elicitation were taken into consideration. Note that some of the false CS detections during acquisition blocks were due to PN responses to the US, which were absent during extinction. A paired-sample T-test revealed a significant difference in CS-FPRs between acquisition and extinction blocks ($t(8) = 5.1$, $P < 0.001$). To allow for better comparison of signal variability during acquisition and extinction, CS-FPR was corrected to disregard false CS detections resulting from the PN's response to the US (inset in b; $t(8) = -0.5$, $P > 0.6$). Paired-sampled T-tests revealed no significant differences between acquisition and

extinction blocks in CS-TPP ($t(8) = 1.7$, $P > 0.12$), or US-FPR ($t(8) = -0.2$, $P > 0.8$). In addition, no significant correlations between period and CS-TPP, US-TPP, US-FPR or corrected CS-FPR were observed (Pearson's correlation, all Ps > 0.3).

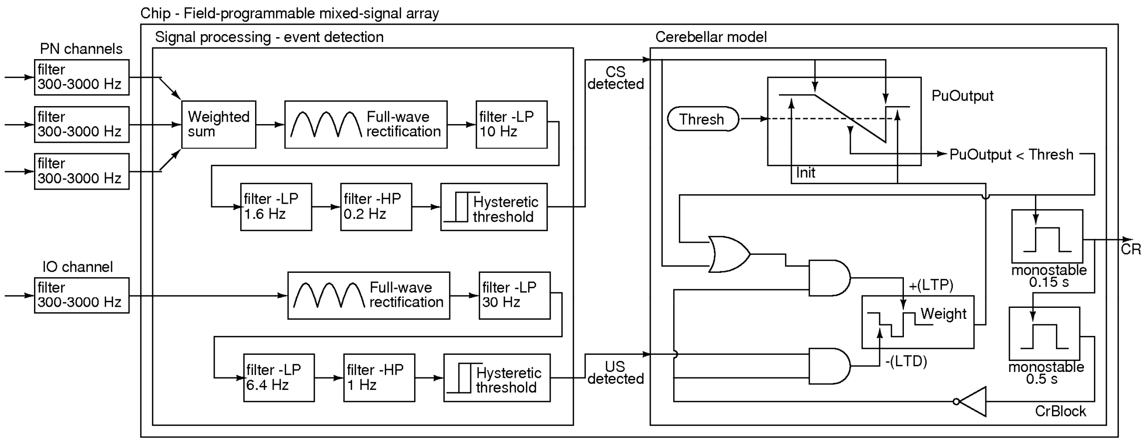

Supplementary Figure 7: Chip design. Schematic of computational pathway implemented in parallel on field-programmable mixed-signal array chip, using a combination of digital and analogue primitives, particularly switched capacitor circuits, as previously described[24]. Electrodes were connected to a standard amplification system (MCP-plus, Alpha-Omega, Israel) which applied 10000 gain and Butterworth filters: 2-pole high-pass at 300 Hz; 4-pole low-pass at 3000 Hz; these signals were inputs to the chip. PN signals were summed with weights chosen in a previous parameterization. Then both the PN and IO pathway applied full-wave rectification, followed by band-pass filtering and hysteretic thresholding to produce a digital signal representing detection of stimulus events (CS and US). The band-pass filtering was constructed as a series of single pole filters - two low pass followed by one high pass. Having two consecutive low-pass stages helped to avoid extreme capacitor ratios, given limited programmable resources. The cerebellar model took CS and US events to trigger both the ramping of a voltage representing the output of PU, and long-term plasticity rules. In particular, the crossing of PU output below a threshold resulted in the production of an output pulse which was used by a downstream stimulator to implement the CR eyeblink. The plasticity rules were implemented as digital logic which then incremented and decremented a binary-encoded weight variable, which was then passed through a digital to analog converter, to provide a voltage (Init) with which the PU output was initialized after each CS.
